# Euler-Lagrange Prediction of Diesel-Exhaust Polydisperse Particle Transport and Deposition in Lung: Anatomy and Turbulence Effects

**DOI:** 10.1038/s41598-019-48753-6

**Published:** 2019-08-27

**Authors:** Mohammad S. Islam, Suvash C. Saha, Tevfik Gemci, Ian A. Yang, Emilie Sauret, Zoran Ristovski, Y. T. Gu

**Affiliations:** 1 School of Mechanical and Mechatronic Engineering, University of Technology Sydney (UTS), 15 Broadway, Ultimo, NSW 2007 Australia; 2 School of Chemistry, Physics & Mechanical Engineering, Queensland University of Technology (QUT), 2 George Street, GPO Box 2434, Brisbane, QLD 4001 Australia; 3 Validation Engineer Specialist, B. Braun Medical Inc., 2525 McGaw Avenue, Irvine, CA USA; 4 Department of Thoracic Medicine, The Prince Charles Hospital, Metro North Hospital and Health Service, and Faculty of Medicine, The University of Queensland, Brisbane, Australia

**Keywords:** Biomedical engineering, Computational science

## Abstract

In clinical assessments, the correlation between atmospheric air pollution and respiratory damage is highly complicated. Epidemiological studies show that atmospheric air pollution is largely responsible for the global proliferation of pulmonary disease. This is particularly significant, since most Computational Fluid Dynamics (CFD) studies to date have used monodisperse particles, which may not accurately reflect realistic inhalation patterns, since atmospheric aerosols are mostly polydisperse. The aim of this study is to investigate the anatomy and turbulent effects on polydisperse particle transport and deposition (TD) in the upper airways. The Euler-Lagrange approach is used for polydisperse particle TD prediction in both laminar and turbulent conditions. Various anatomical models are adopted to investigate the polydisperse particle TD under different flow conditions. Rossin-Rammler diameter distribution is used for the distribution of the initial particle diameter. The numerical results illustrate that airflow rate distribution at the right lung of a realistic model is higher than a non-realistic model. The CFD study also shows that turbulence effects on deposition are higher for larger diameter particles than with particles of smaller diameter. A significant amount of polydisperse particles are also shown to be deposited at the tracheal wall for CT-based model, whereas particles are mostly deposited at the carinal angle for the non-realistic model. A comprehensive, polydisperse particle TD analysis would enhance understanding of the realistic deposition pattern and decrease unwanted therapeutic aerosol deposition at the extrathoracic airways.

## Introduction

The airborne particles from different natural and man-made sources, and the pharmaceutical particles from different drug delivery tools, exhibit a highly complex size distribution. The complex size distribution of the respirable aerosol particles determines the deposition location in the respiratory system^1^.

Inhaled particle deposition in the respiratory tract is caused mainly by inertial impaction, Brownian diffusion, gravitational sedimentation, and interception^2^. Also, aerosol particle deposition in the bifurcating airway is governed largely by its size^3–7^. Particulates of diameter >5 *µm* are deposited in extrathoracic airways, whereas particles from 1 *µm*–5 *µm* are deposited in the conducting airways^8,9^. Particulates of size <1 *µm* are deposited in deeper airways, such as the alveoli region, and in peripheral airways^9,10^. Micro-particles less than 0.5 *µm* are initially deposited in the human lung by Brownian diffusion^5,11^, while larger particles are deposited by sedimentation and inertial impaction^12^. Polydisperse particles from various atmospheric sources are inhaled through nasal and oral airways. Almost all of the published studies assume particles of different sizes for human lung modelling. However, most of the literature did not consider the polydisperse particle for their study. The understanding of the polydisperse aerosol particle TD for the extra-thoracic airways of the lung is the primary step to; therefore, an all-inclusive polydisperse particle TD study is important for a comprehensive examination of particle TD in the pulmonary airways.

A variety of *in silico* and *in vivo* models have been designed for particle TDs in the extrathoracic and intrathoracic airways^13–21^. Most of these studies utilized monodisperse aerosol particles to look into the particle TD in the bifurcating airways. They showed the Monte Carlo modelling of aerosol particle deposition in a stochastic lung model^22–24^. This series of studies reports the monodisperse particle deposition fraction sensitivity in various regions for different deposition parameters. Both typical single-path models^25,26^ and multiple-path models^27^ were used to perform the monodisperse particle transport in the pulmonary airways. A comparison study shows that both smaller and larger diameter particle depositions vary measurably in the central airways of the lung^28^. In addition, a low Reynolds-number (LRN) *k*-ω model shows a higher non-uniform deposition pattern for monodisperse micro-particles. The occupational and ambient properties of the aerosol particles are polydisperse^29^ and have been related to adverse pulmonary health effects^30^.

Hygroscopic properties (changes of size due to water absorption, morphology, chemical composition, and reactivity) of polydisperse particles influence the overall deposition pattern in the human lung, and experiments have shown different deposition probabilities of hygroscopic aerosol compared to non-hygroscopic aerosol^31^. Ferron, *et al*.^32^ reported the error estimation of hygroscopic polydisperse aerosol deposition in the pulmonary airways, which is less than 10% for monodisperse particles.

An *in vivo* model of submicron particle (d16-d84) TD in a child’s lung showed 72% ± 17% radioactive polydisperse aerosol deposition in the extra-thoracic upper airways^33^. The study showed that particle diameters from 0.15 µm–0.5 µm are mostly (84% ± 4%) deposited in the thoracic region. Particle diameters from 0.25 µm–1 µm show 49% ± 8% deposition in the extra-thoracic region and 51% ± 8% in the thoracic region. An artificial neural network-based prediction gave a more accurate prediction of regional deposition, and the typical error was less than around 0.025%^34^. Recently, a Euler-Lagrange-based CFD study used a human Zygota5 model for better prediction of polydisperse aerosol in the human lung^35^. This study indicated that the CFD surface meshes and the ridge of the experimental casts were physically similar and received similar deposition. There are no experimental or CFD studies that have been conducted for the comprehensive polydisperse particle TD in a realistic lung model as a function of different deposition parameters. The precise understanding of the lung anatomical model effects on polydisperse particle TD is important for better health risk assessment. This study used three different triple bifurcations (G0-G3) anatomical models, a CT-based realistic lung model, a symmetric, and an asymmetric model, to predict polydisperse aerosol particle TDs in the upper airways. The turbulence dispersion effects on polydisperse micro- particle TD in the upper airways were investigated. A comprehensive analysis was conducted for the polydisperse particle TD in the right and the left lung.

## Geometry Generation

**Figure 1:**
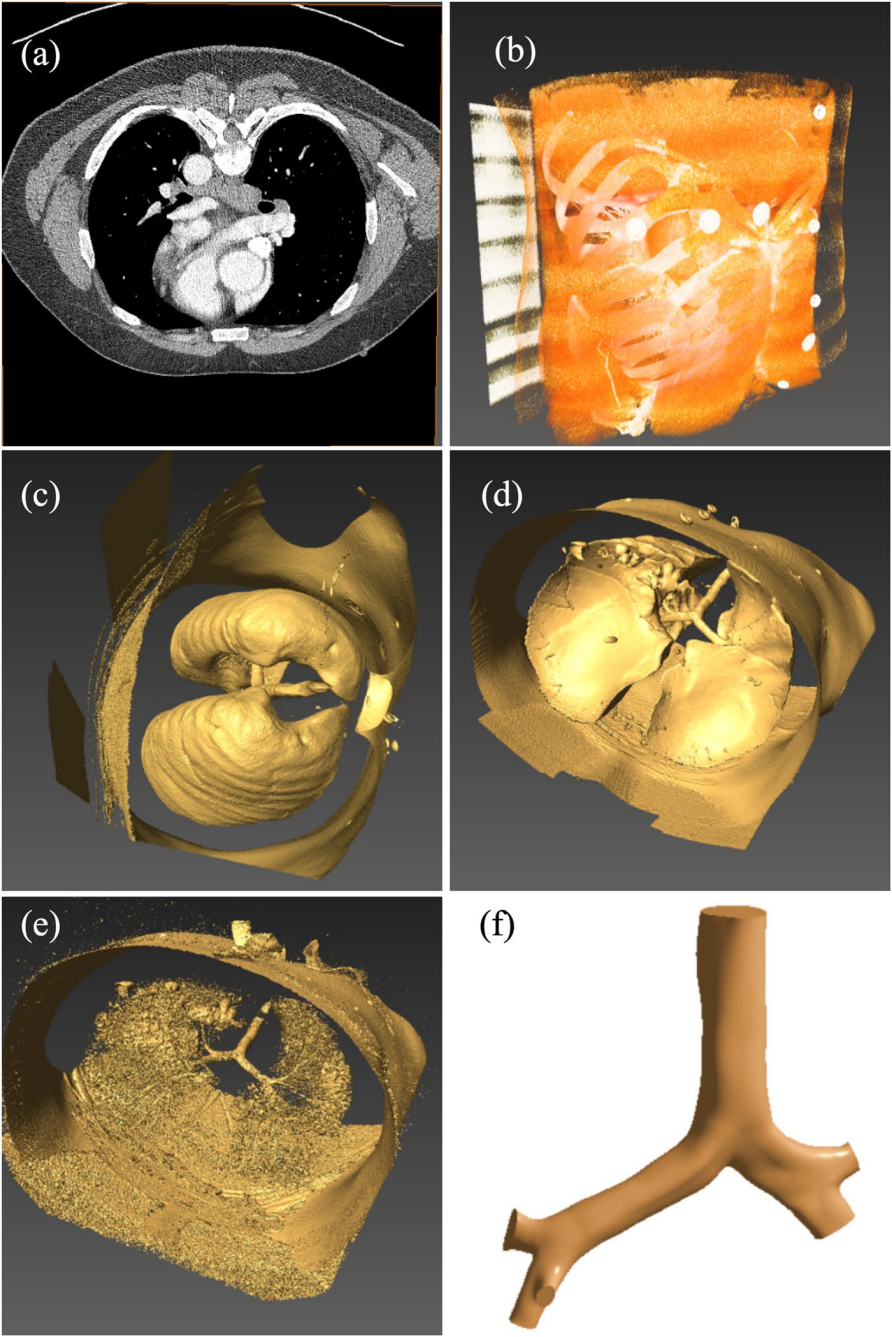
Geometry generation from the CT-based DiCom images of a healthy adult, (**a**) visualization of the raw DiCom images, (**b**) airway tree with skeleton, (**c**) 3D lung airway with right and left lobe, (**d**) bifurcation views with left and right lung, (**e)** constructed model with missing surface and **(f**) final 3-D model for the upper airways.

A reconstructed 3D anatomical model of the upper airways has been developed from the realistic CT-images of a 51-year healthy male. An appropriate ethical clearance has been obtained from the review committee of the Prince Charles Hospital. Figure 1 shows the different steps to construct the 3D anatomical model. Different visualization and computer-aided software’s are used for geometry construction. Visualization software AMIRA is used to visualize the raw CT-images and volume rendering purpose. The CT DiCom images are imported to AMIRA (geometry generation software) and creates ortho-slice. Figure 1(a,b) show the initial view of the CT images and the chest skeleton. Once the raw materials and chest structure are visible, isosurface is created for the clear branching pattern. For better visualization of the right and the left lobe branches, raw materials of the left lobe and right lobe are cleared by setting an appropriate threshold. The threshold set up for removing the raw material will be different for every CT-data because of the different image resolution. Figure 1(c) shows the 3D view of the airways. Figure 1(d) shows the 3D view of the airway along with the different lobes. The threshold setup process creates some missing surface (Fig. 1(e)) at the upper airways due to the resolution variation of the CT-images. The visible part of the branching pattern is imported to the Geomagic software, and the missing surfaces are reconstructed. Finally, the 3D model is imported to the Solidworks and prepared the ANSYS supported version of the 3D model (Fig. 1f).

During the 3D model construction, there was some missing surface (Fig. 1(e)), and Geomagic software is used to reconstruct the missing surfaces. Finally, the constructed model is imported to SolidWorks for further processing, and final 3D model is constructed, which is shown in Fig. 1(f).

The details airway dimensions of the CT-based airway model are calculated, and Table 1 shows the airway diameter of different generations and corresponding branches length.

**Table 1:** Dimensions of the realistic CT-based anatomical model (in mm).

Trachea Length	63.42
Inlet Hydraulic Diameter	18.6258
G-1 Right Branch Length	31.40
Right Branch Hydraulic Diameter	14.6325
G-1 Left Branch Length	58.87
Left Branch Hydraulic Diameter	13.684
G-2 Right Branch Length (Right Lung)	12.75
G-2 Right Branch Diameter (Right Lung)	8.045
G-2 Left Branch Length (Right Lung)	13.98
G-2 Left Branch Diameter (Right Lung)	11.45
G-2 Right Branch Length (Left Lung)	12.55
G-2 Right Branch Diameter (Left Lung)	9.82
G-2 Left Branch Length (Left t Lung)	10.43
G-2 Left Branch Diameter (Left Lung)	9.99
G-3 Right Branch Length (Left Lung)	7.26
G-3 Right Branch Diameter (Left Lung)	5.83
G-3 Left Branch Length (Left t Lung)	11.04
G-3 Left Branch Diameter (Left Lung)	7.48

**Figure 2:**
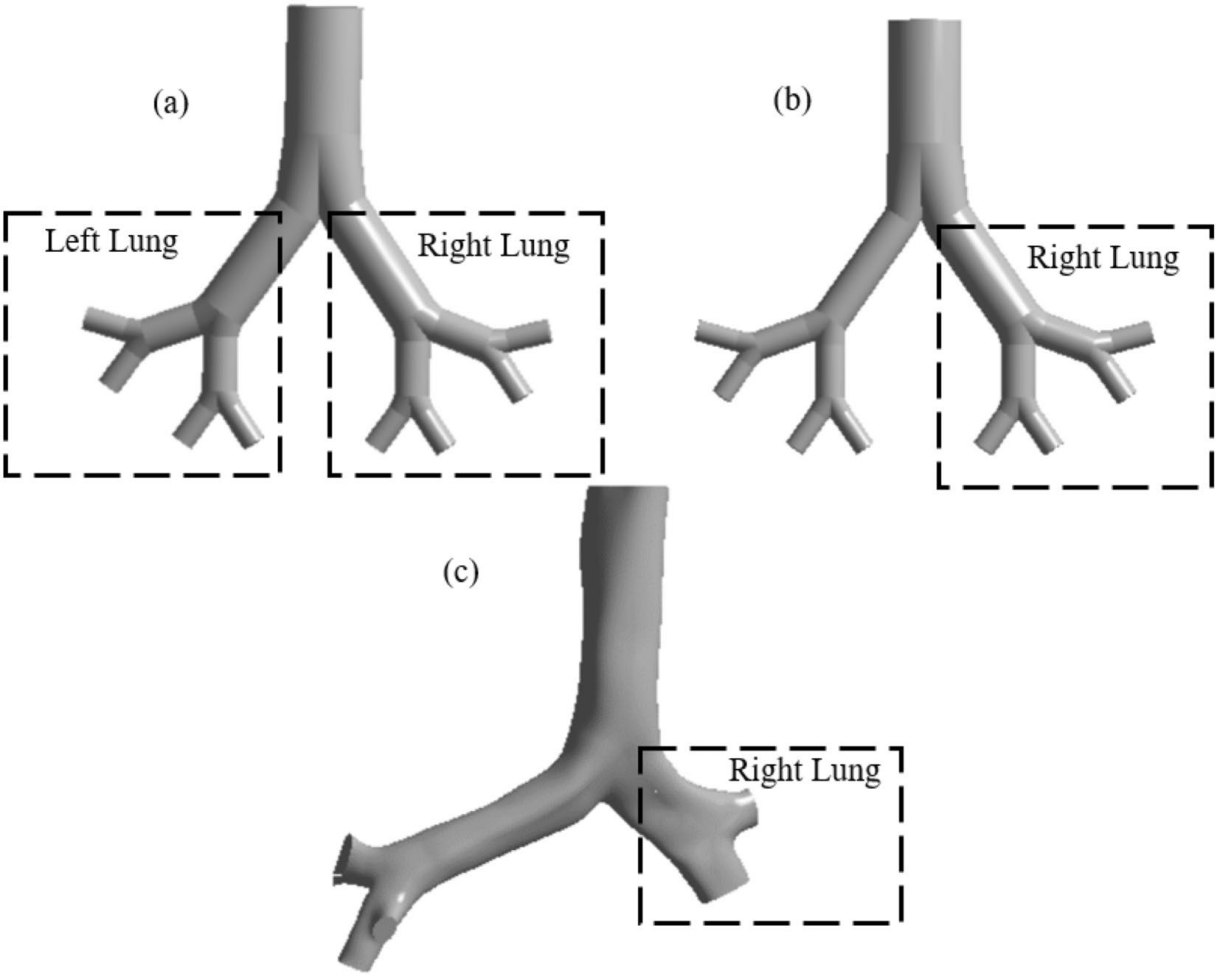
Final 3-D anatomical model up to first three-generation; (**a**) non-realistic symmetric, (**b**) non-realistic asymmetric, and (**c**) realistic CT-based.

A highly asymmetric realistic model from CT-Scan DiCom images, a symmetric triple bifurcation model from Weibel’s lung model and an asymmetric smooth surface model is developed for the present study. Figure 2(a–c) show the realistic CT-based, symmetric, and asymmetric lung model, respectively. The detail calculated dimensions for the CT-based realistic model is shown in Table 1. For the symmetric model, Weibel’s lung model dimensions are used. The anatomical branching diameter of the right lung and the left lung are different. The right lung diameter is higher than the left lung, while the right lung is shorter than the left lung. However, for Weibel’s model, the right lung and the left lung are symmetric.

### Numerical methods

The general mass and momentum equations are used for the flow field calculations. The governing equations for the mass and momentum are used as:

1 $$\frac{\partial \rho }{\partial t}+\nabla \cdot (\rho \overrightarrow{{\rm{v}}})={S}_{m}$$

where *S*_*m*_ is the source term.

2 $$\frac{\partial }{\partial t}(\rho \overrightarrow{{\rm{v}}})+\nabla \cdot (\rho \overrightarrow{{\rm{v}}}\overrightarrow{{\rm{v}}})=-\,\nabla p+\nabla \cdot (\mu [(\nabla \overrightarrow{{\rm{v}}}+\nabla {\overrightarrow{{\rm{v}}}}^{{\rm{{\rm T}}}})-\frac{2}{3}\nabla \cdot \overrightarrow{{\rm{v}}}I])+\rho \overrightarrow{g}+\overrightarrow{F}$$

where, the static pressure of the fluid is *p*, gravity-induced body force is $\rho \overrightarrow{g}$, and the body force generated by the external force is defined as $\overrightarrow{F}$. The Large Eddy Simulation (LES) model is employed to investigate turbulence effects on micro-sized polydisperse particle TDs in the upper airways. The Smagorinsky-Lilly subgrid-scale (SGS) model is used to calculate the smaller eddies based on the airflow modelling of Gemci *et al*.^36^. Some other turbulent models, including the k-ω and k-${\rm{\varepsilon }}$ model, are used to compare the particle transport results with the LES turbulent model. SIMPLE scheme^35^ and the second-order pressure, turbulent kinetic energy, upwind momentum, and specific dissipation rate discretization are used. Euler-Lagrange based Discrete Phase Model (DPM) is used for particle TD. Particle is treated as the disperse phase. Lagrangian reference frame and corresponding particle transport equation is solved. The particle transport equation can be written as;

3 $$\frac{d{\overrightarrow{u}}_{p}}{dt}={F}_{D}(\overrightarrow{u}-{\overrightarrow{u}}_{p})+\frac{\overrightarrow{g}({\rho }_{p}-\rho )}{{\rho }_{p}}+\overrightarrow{F}$$

where additional acceleration term is $\overrightarrow{F}$, the drag force term is ${F}_{D}(\overrightarrow{u}-{\overrightarrow{u}}_{p})$. Drag force of the spherical particle is the domain force leading the particles and can be defined as;

4 $${\overrightarrow{F}}_{D,i}=\frac{1}{2}{C}_{D}\frac{\pi {d}_{p,i}^{2}}{4}\rho ({\overrightarrow{v}}_{p,i}-\overrightarrow{v})|{\overrightarrow{v}}_{p,i}-\overrightarrow{v}|$$

where *C*_*D*_ is the drag coefficient, *d*_*p*_ is the diameter of the particle, and ${\overrightarrow{v}}_{p}$ is the particle velocity.

Velocity inlet and pressure outlet conditions are employed for the upper airway model. A parabolic profile is used at the inlet^37^. Normal to boundary velocity specification method is used for the velocity inlet. A zero-pressure is used at all outlets. In reality, there should be a small pressure difference at the outlets of a whole lung model. This study considered only the first three-generation, and zero pressure outlet conditions are employed at all outlets. The particles are injected from the inlet surface, and each facet of the inlet surface injected a single particle. The particles size distribution is used as non-uniform, and particles are injected using the face normal directions. All particles are injected at once. A total of 12000 aerosol particles were released through the inlet surface, with particle density of 1100 kg/m^3^. Different groups of particles were used to test the convergence of the local particle deposition and found that the difference of the local deposition for 12000 particles is negligible (less than 1%) with other groups of particles. The Rossin-Rammler diameter distribution technique was used to introduce the polydisperse particles into the present model^38^. A wide range of particle sizes (1 μm ≤ *d*_*p*_ ≤ 10 μm) was considered for the present model. Note that different diameter aerosol particles were randomly released from the lung inlet surface. The wall surface condition is used as ‘no-slip’^39,40^, and the airway wall was stationary. A ‘trap’ condition was used at the airway wall for particle deposition. The trap conditions mean, if the particle touches the airway wall surface, it will be treated as deposit. Individual particle movement was tracked by using our own code and ANSYS Fluent 17.2 solver was used for the solutions of the governing equations with the help of initial and boundary conditions. An in-house MATLAB code was developed and used for particle deposition concentration calculation. All methods performed in this study were in accordance with the relevant guidelines and regulations.

### Grid test and model validation

**Figure 3:**
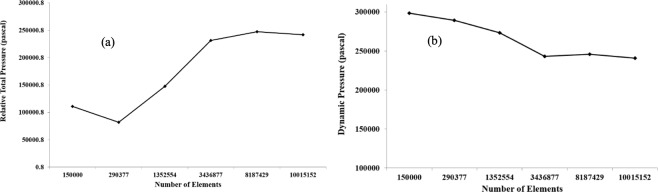
Grid refinement test result for realistic lung model at 60 lpm flow rate; (**a**) relative total pressure and (**b**) dynamic pressure.

An unstructured tetrahedral element was constructed for the triple bifurcation model. The realistic CT-based model exhibited a highly asymmetric branching pattern and complex wall surfaces. To model the complex flow field near the wall, a smooth transitional inflation layer mesh was generated. A fine ten-layer inflation mesh was placed near the airway wall and hexahedral elements were used. A dense tetrahedral mesh was used at the carinal angle of the triple bifurcation model. The fine hexahedral element near the airway wall and the dense tetrahedral mesh element at the carinal angle area are used for the better treatment of the turbulent flow. Depending on the implemented turbulent model, the y^+^ value of the grid refinement was less than 1. A standard grid refinement test was performed for all models against six different number of grids and the refinement test results for the CT-based model are shown in Fig. 3. The pressure values have been plotted at the outlets of the CT anatomical model. The grid refinement test shows the pressure difference for different set of meshes is negligible from 3.44 million cells and the final mesh contains about 3.44 million computational cells.

**Figure 4:**
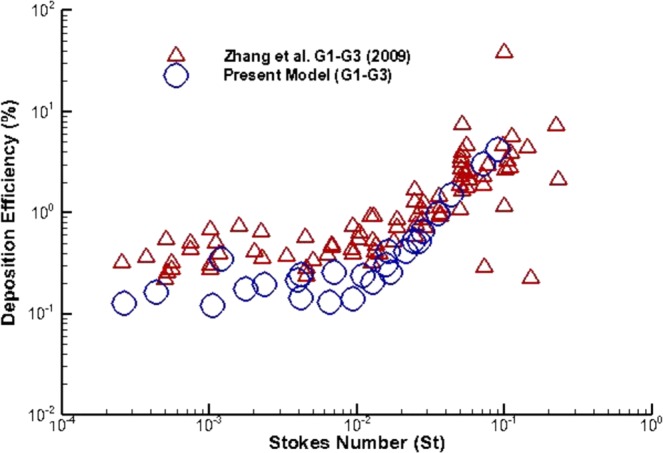
Deposition efficiency comparison of micron particle against Stokes number for a triple bifurcation (Weibel based) anatomical model.

The present CFD study for polydisperse particle TD was validated against available published data. The deposition efficiency (DE) vs. Stokes number has also been calculated and compared. The dimensionless local Stokes number (*S*_*t*_) can be defined as a function of aerosol particle density, diameter of the particle, air speed, viscosity, and diameter of the pipe. The Stokes number can be defined as:

5 $${S}_{t}=\,\frac{\rho {{d}_{p}}^{2}u}{18\mu D}$$

where *ρ* is particle density, particle diameter is *d*_*p*_, viscosity of air is *μ*, and *D* is the pipe diameter. DE comparison results for the first three generations are shown in Fig. 4. The DE is compared with the CFD study of Zhang, *et al*.^41^. The DE against the Stokes number is calculated for LES model. The overall DE comparison shows a good match with the published data for large Stokes number. For lower Stokes number, the DE shows some deviation with the DE of Zhang *et al*.^41^. However, the DE for lower Stokes number shows a good agreement with a couple of specific points of Zhang *et al*.^41^ results. The overall comparison shows a good agreement, which indicate that the CFD model is accurate enough to predict particle TD in bifurcating airways.

## Results and Discussions

**Figure 5:**
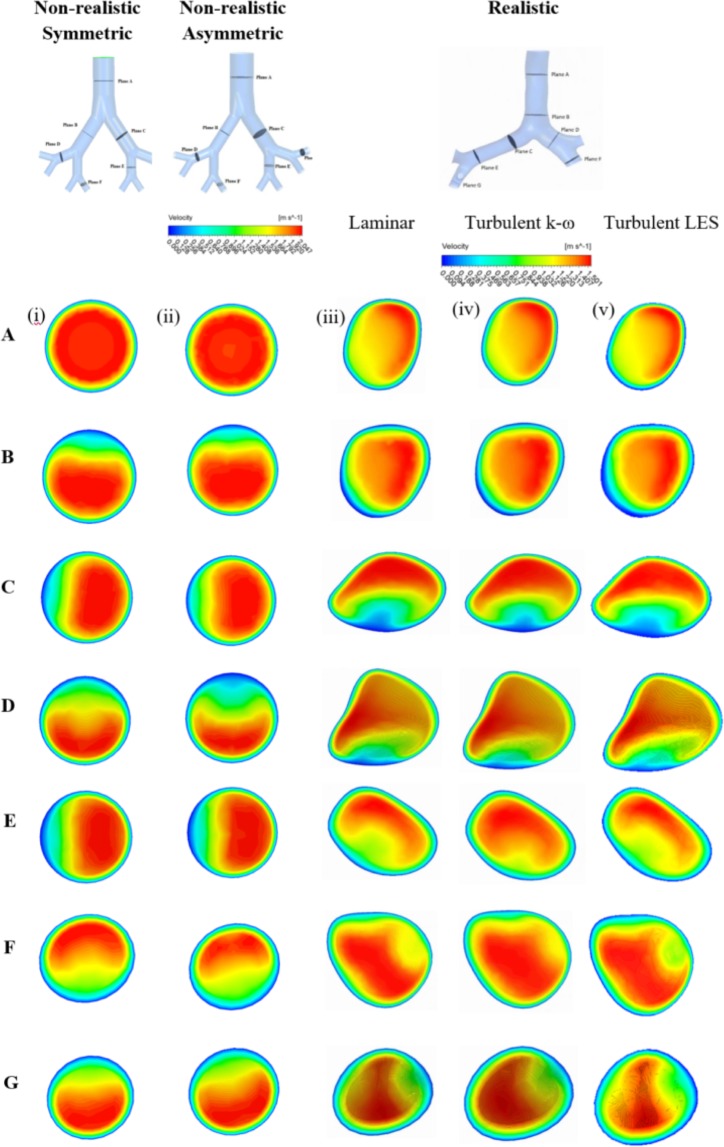
Velocity contour at different selected plane during 15 lpm flow rate; (i) non-realistic symmetric model, (ii) non-realistic asymmetric model, (iii) realistic model-laminar case, (iv) realistic model-turbulent k-ω case, and (v) realistic model-turbulent LES.

A triple bifurcation symmetric Weibel lung model, an idealized asymmetric lung model, and a realistic CT-scan model were considered in the present study. A comprehensive polydisperse particle TD was performed for several flow conditions. Figure 5 reports velocity contours at seven selected planes in a triple bifurcation model for a 15 lpm flow rate. Velocity contours were drawn at seven planes in the non-realistic symmetric, asymmetric, and realistic lung model. The non-realistic symmetric and asymmetric model shows nearly similar velocity contours, except at planes D and E. The right panel in Fig. 5 shows the velocity contour in realistic geometry for the laminar and turbulent conditions. The velocity contours at the randomly selected planes of a CT-based model for 15 lpm flow shows almost similar flow pattern and which eventually indicate that turbulent dispersion for lower flow rate is insignificant.

**Figure 6:**
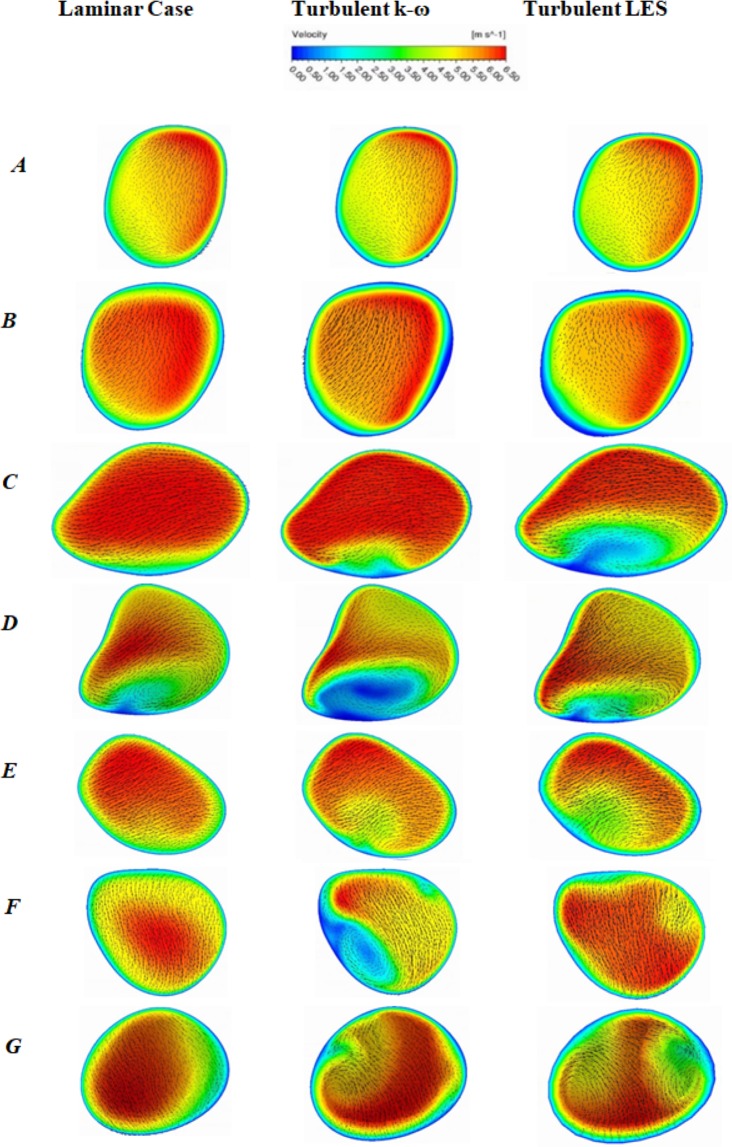
Velocity contour at different selected plane during 50 lpm flow rate for realistic anatomical model.

The airflow pattern of a realistic human lung could be locally turbulent at upper airways at medium and higher inspiratory flow rates. The velocity contours at the selected planes are drawn for 50 lpm flow rate and which is presented in Fig. 6. The overall velocity contours show strong turbulent fluctuation at the selected planes of the right lung than the left lung. For the realistic model, vortices were generated at different planes because of the strong change of cross-sectional area. Turbulence intensity, a highly complicated branching pattern and a centrifugally induced pressure gradient also influenced the velocity pattern at various planes of the bifurcating branches.

The total flow rate distribution percentage in the right and the left lung in a non-realistic and realistic triple bifurcation model is shown in Table 2. The findings of the present study are compared with the study of Horsfield, *et al*.^42^ and Cohen, *et al*.^43^. The flow rate distribution in the realistic CT-based anatomical model shows a greater flow distribution in the right airways, compared to the non-realistic model. The highly asymmetric anatomical model of the realistic lung is the primary reason for the higher flow distribution in the right lung.

**Table 2:** Total flow rate distribution in a triple bifurcation airway model.

	15 lpm	25 lpm	50 lpm	Cohen, *et al*.^43^	Horsfield, *et al*.^42^
Right Lung Realistic	62.84	62.44	62.27	59	54.6
Left Lung Realistic	37.16	37.56	37.73	41	45.4
Right Lung Non-Realistic	58.51	58.45	58.18	—	—
Left Lung Non-Realistic	41.48	41.54	41.82	—	—

**Figure 7:**
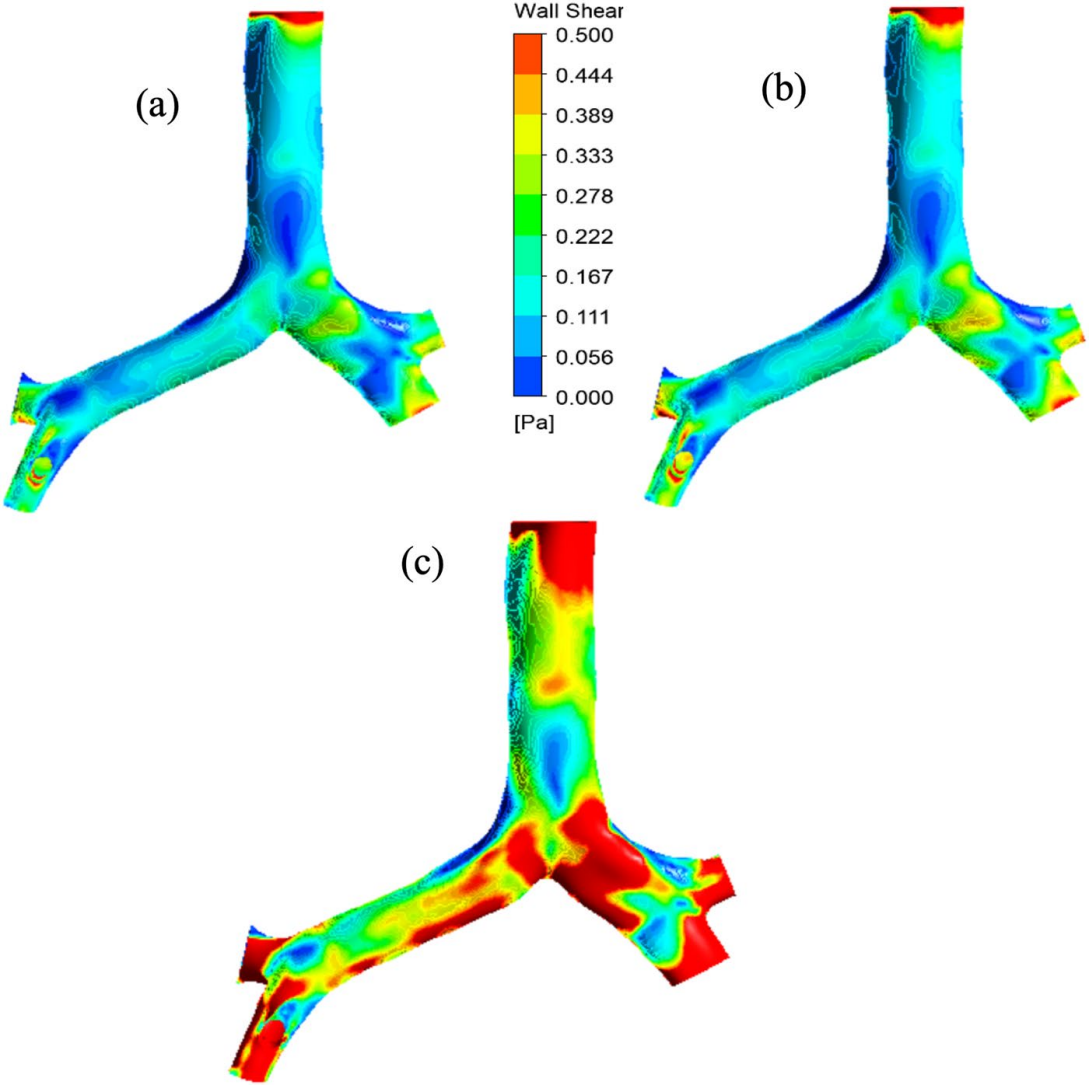
Wall shear for the realistic model at (**a**) 50 lpm flow rate *k*-ω model, (**b**) 50 lpm flow rate LES model, and (**b**) 60 lpm flow rate LES model.

The wall shear for the CT-based realistic model is calculated for 50 lpm and 60 lpm flow rate. The calculated wall shear is presented for a user-defined range of value. Figure 7(a) presents the wall shear for *k*-ω turbulent model and Fig. 7(b) illustrates the wall shear for LES turbulent model at 50 lpm flow rate. At 50 lpm flow rate, wall shear at the top of the inlet surface is maximum. The wall shear at the terminal airways of the realistic model is also found maximum for 50 lpm flow rate. The overall wall shear for *k*-ω turbulent model and LES turbulent model at 50 lpm flow rate is found almost similar. At higher flow rate (60 lpm), the wall shear contour shows a complex shear pattern for CT based model. Maximum wall shear value is found at the different positions of the realistic anatomical model.

**Figure 8:**
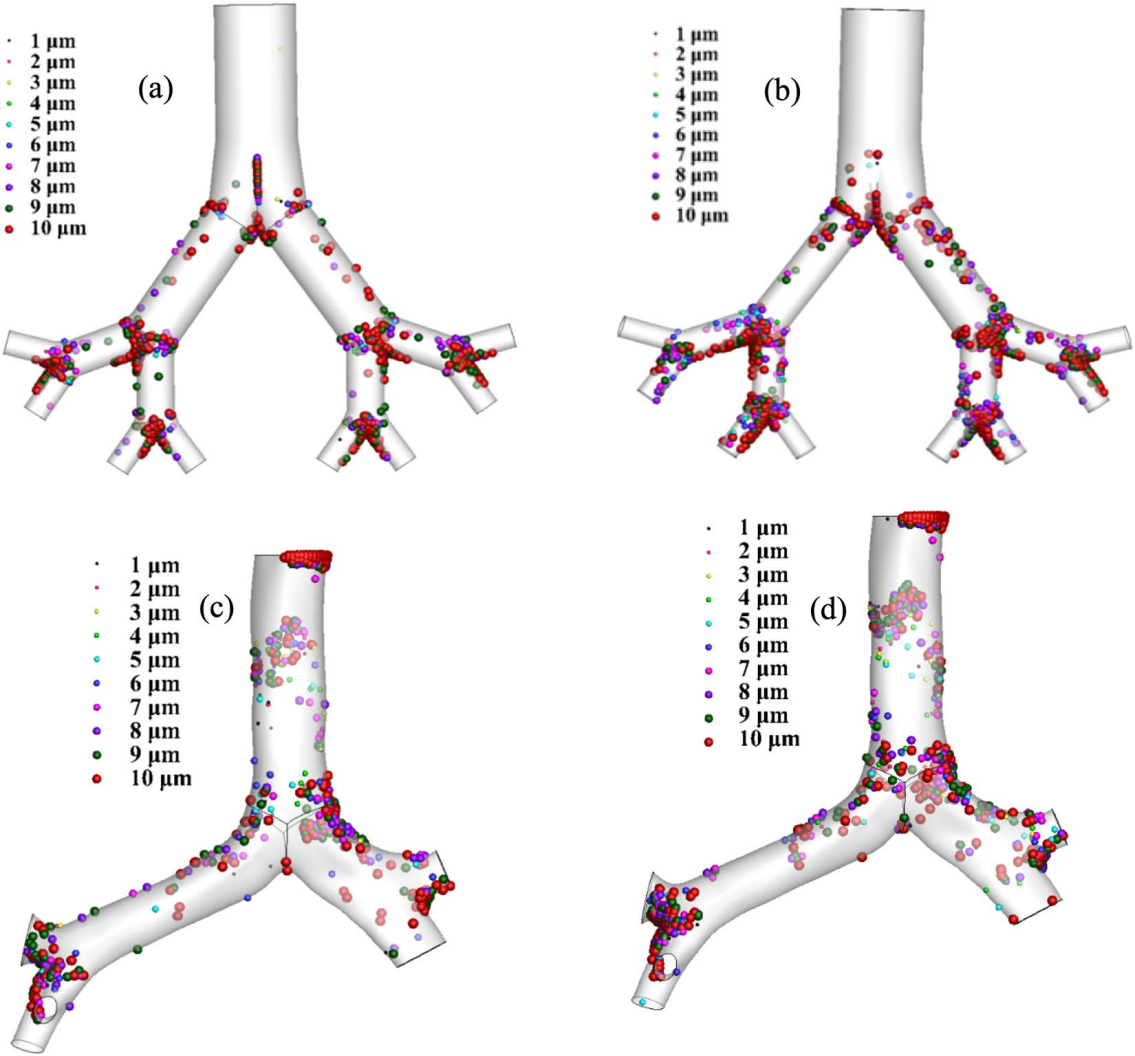
Polydisperse aerosol particle deposition scenario at 50 lpm, (**a**) symmetric lung model (**b**) asymmetric lung model, (**c**) realistic- turbulent *k*-ω model, and (**d**) realistic-LES model.

The respiratory deposition scenario of a polydisperse particle in a triple bifurcation of the non-realistic and realistic lung model is shown in Fig. 8. A wide range of micro-particles (1 μm ≤ *d*_*p*_ ≤ 10 μm) are considered and the particle diameter is defined by assorted colours. The overall deposition pattern in a non-realistic symmetric and asymmetric triple bifurcation model reports no aerosol particle deposition at the trachea. The deposition pattern illustrates that the bifurcation region (carinal angle region) is the deposition hot spot (DHS) in the non-realistic model. However, the CT-based realistic model shows a different deposition scenario. In the CT model, a significant number of aerosol particles are trapped in the trachea, and the airway wall, compared to the carinal angle region. The respiratory deposition pattern for different turbulent models are investigated. The deposition pattern for *k*-ω turbulent model and LES turbulent model at 50 lpm flow rate illustrate a negligible deposition variation. The overall deposition pattern in the realistic model demonstrates that both the airway wall and the carinal angle are significantly affected. Moreover, the overall deposition pattern in both models reports that fewer smaller particles are trapped than the larger diameter particles.

Micro-particle inertia and the geometrical asymmetricity of the airway model influences the overall deposition. In the non-realistic model, most aerosol particles are trapped at the bifurcation area, because of the higher inertia of the polydisperse micro-particle. The airway wall surface of the non-realistic model is smooth; the only obstruction is in the bifurcation area. During their movement, micro-particles follow the air stream; larger diameter particles cannot follow the air path due to its higher inertia when the particles face the strong deviation from the previous path at the carinal angle region. Because of the higher inertia and asymmetricity at the carinal angle, a substantial number of particles are trapped at the carinal angle area of the non-realistic model. Due to the symmetric and smooth tracheal wall, no particles are trapped at the tracheal area of the non-realistic model. However, the present CT-based model exhibits a highly asymmetric tracheal and bronchial wall throughout the bifurcation. As a result, a large number of aerosol particles are set down at the trachea and airway wall throughout the geometry. Due to the lower inertia of the smaller diameter particles, fewer smaller diameter particles are trapped at the upper airways.

**Figure 9:**
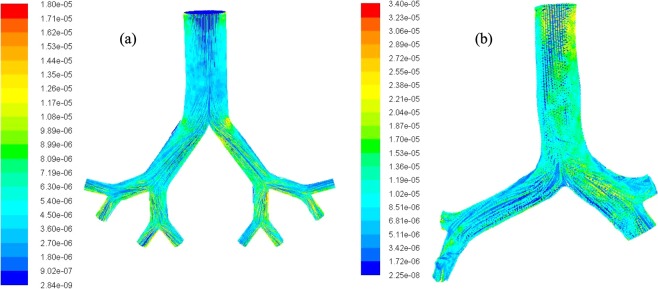
Polydisperse particle pathline at 50 lpm flow rate coloured by subgrid turbulent viscosity, (**a**) non-realistic model and (**b**) realistic model.

The pathlines for the polydisperse particles are calculated at 50 lpm flow rates. Figure 9 shows the pathlines for non-realistic and realistic model coloured by subgrid turbulent viscosity. At non-realistic model, particles pathlines are straightforward throughout the branching pattern except the bifurcation areas. The viscosity at the bifurcating area is higher than the airway branching area. Non-realistic airway model is geometrically simple with smooth wall surface however, the bifurcation zone exists a sharp bend. Figure 9(a) shows the particle trajectories at the bending area are complex than the remaining branching area. The subgrid turbulent viscosity at the bifurcation area is maximum for the non-realistic model. The general deposition visualization figure (Fig. 8) depicts that micro diameter aerosol particles are mostly trapped at the bifurcation area for the non-realistic model. The particle pathlines of Fig. 9(a) support the general deposition pattern at the non-realistic model. For realistic CT-based model, the particle pathlines show maximum turbulent viscosity at the upper part of the trachea and the right bronchi of the first bifurcation. The overall particle pathlines for CT-based model shows more complex transition than the non-realistic model.

**Figure 10:**
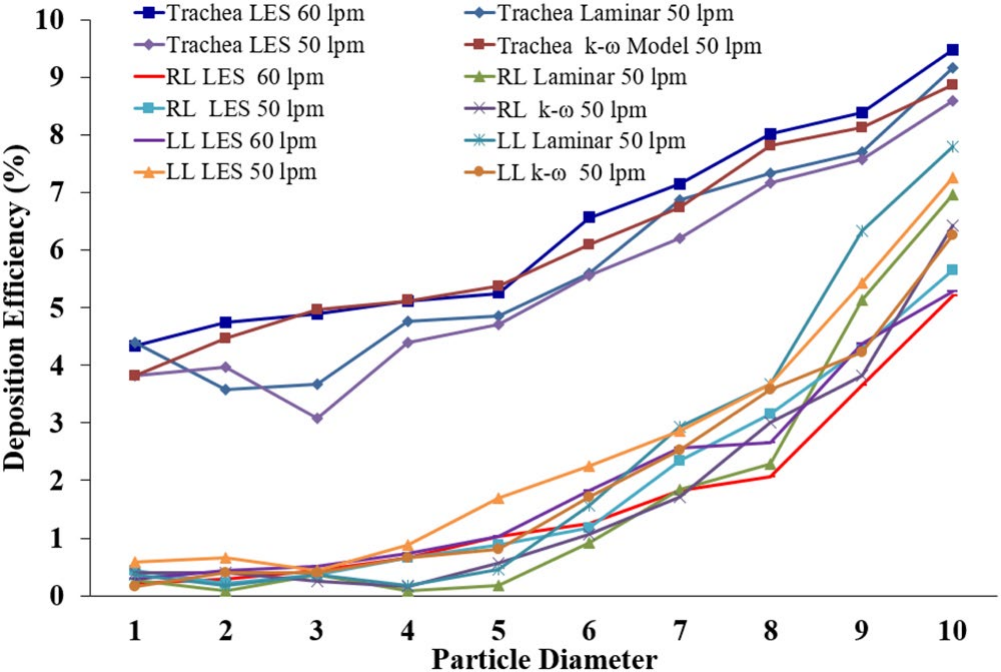
Polydisperse particle DE in different region of the realistic lung model at 50 lpm and 60 lpm.

The DE of different diameter particles was calculated based on total deposition, which is shown in Fig. 10. Laminar and turbulent cases are performed for different flow rates. The DE plot shows that DE in the tracheal wall is pointedly greater than the right and left lung at 50 lpm flow rate. Of the total deposition, 61.43% of the polydisperse particles are trapped at the tracheal wall for the turbulent case, whereas 38.57% of polydisperse particles are deposited at the both right and left lung. At 60 lpm, 63.98% of the polydisperse particles are trapped at the tracheal wall and 36.02% particles are deposited at both lungs. At laminar case, 57.98% of the polydisperse aerosols are trapped at the trachea and 42.02% particles are trapped in the airway branches. The overall DE at the tracheal wall due to turbulent dispersion is higher than the laminar case. The polydisperse particle DE for laminar case shows that only 5.05% of 1-µm particles are deposited, but 76.14% of particles with a diameter from 6–10 µm were deposited at 50 lpm. At 50 lpm flow rate, the DE of the different diameter particle at the right lung and the left lung for LES turbulence model is found higher than the DE of k-ω model. The DE plot also reports that very few smaller diameter particles (*d*_*p*_ ≤ 4 μm) are deposited in the both right and left lung, indicating that smaller particles escaped through the outlets of the present model. The particles are expected to enter into the fourth generation of a continuous whole lung model. The details DE percentage of the different diameter particles during laminar and different turbulent cases are investigated at a different flow rate (Table 3). The overall DE comparison for laminar and turbulent cases show a higher percentage of the particles are trapped at the trachea than the left and the right lung at 50 lpm. The highly asymmetric structure of the tracheal wall, inertial impaction, and local turbulent fluctuation lead higher deposition at the tracheal wall than the right and the left lung. The flow at the extrathoracic airways become locally turbulent at the flow rate greater than 30 lpm and the turbulent fluctuation persist up to the tracheal wall^44^. At 60 lpm flow rate, the turbulent fluctuation at the tracheal wall region and microparticle inertia influence the particle transport and deposition. The comprehensive zone-specific DE analysis for laminar and turbulent cases will improve the understanding of the particle deposition data in the upper lung.

**Table 3:** Polydisperse particle DE comparison at various region of the realistic airways for different flow rate.

Particle Size (µm)	Trachea			Right Lung			Left Lung		
	LES 50 lpm	k-ω Model 50 lpm	LES 60 lpm	LES 50 lpm	k-ω Model 50 lpm	LES 60 lpm	LES 50 lpm	k-ω Model 50 lpm	LES 60 lpm
1	3.81511	3.824247	4.3447	0.44020	0.406835	0.2189	0.58694	0.162734	0.2919
2	3.96184	4.475183	4.7445	0.22010	0.406835	0.2919	0.66030	0.406835	0.4379
3	3.08195	4.963385	4.8905	0.36683	0.244101	0.4379	0.44020	0.406835	0.5109
4	4.40205	5.126119	5.1094	0.66030	0.162734	0.6569	0.88041	0.650936	0.7299
5	4.70205	5.37022	5.2554	0.88041	0.569569	1.0218	1.68745	0.81367	1.0218
6	5.56236	6.102522	6.5693	1.17388	1.057771	1.2408	2.24123	1.708706	1.8248
7	6.21614	6.753458	7.1532	2.34776	1.708706	1.8248	2.86133	2.522376	2.5547
8	7.17644	7.811229	8.0291	3.15480	3.010578	2.0656	3.66837	3.580146	2.6496
9	7.58349	8.136697	8.3941	4.29552	3.824247	3.6496	5.4292	4.231082	4.3795
10	8.59053	8.868999	9.4890	5.64930	6.42799	5.2043	7.26339	6.265256	5.2854

**Figure 11:**
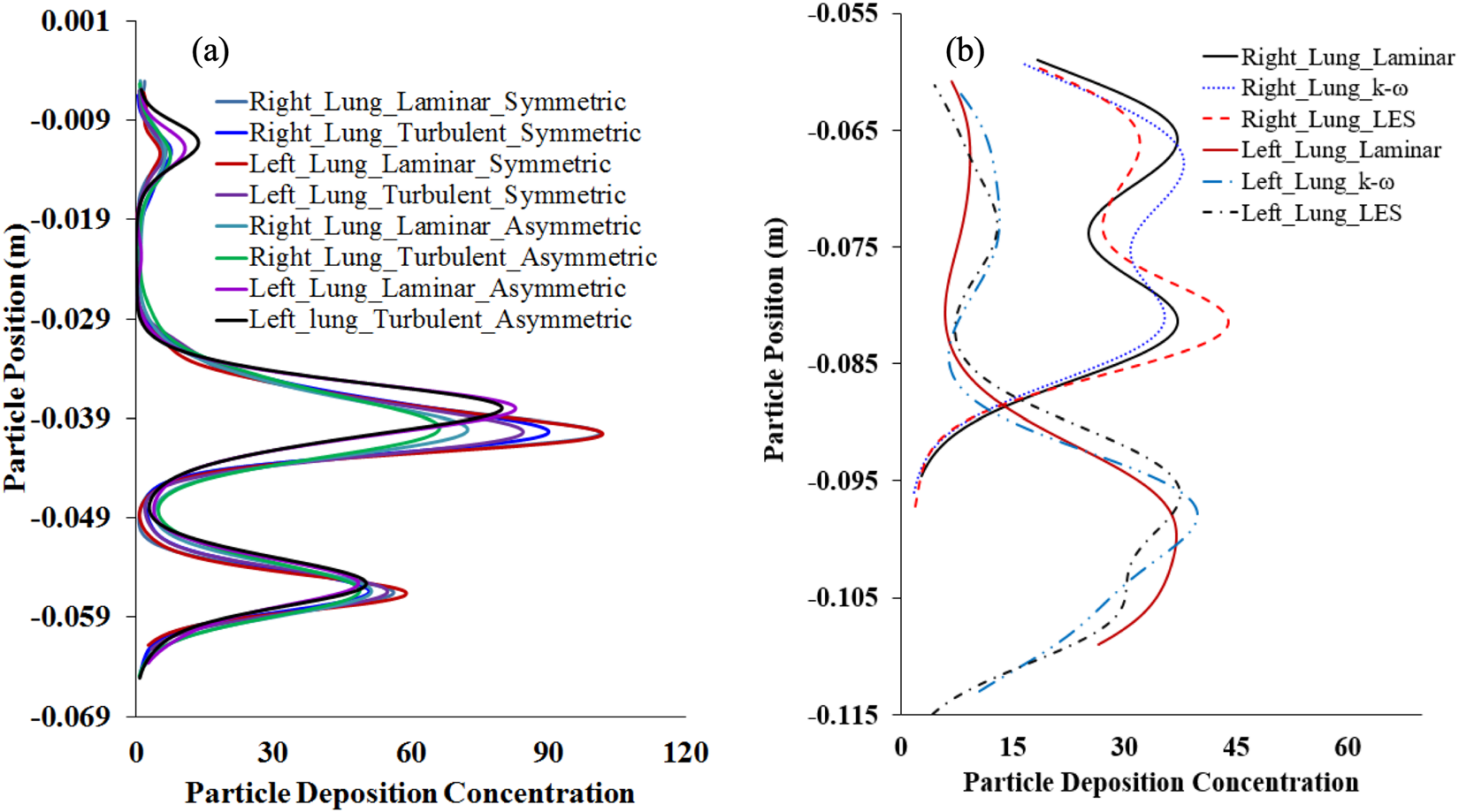
Particle deposition density comparison for different lung models at 50 lpm flow rate, (**a**) non-realistic symmetric and asymmetric model, and (**b**) realistic CT-based model.

The deposition density comparisons in the non-realistic and realistic models are reported in Fig. 11. Figure 11(a) reports the trapped particle density comparison in the asymmetric and the symmetric model for laminar and turbulent cases. The overall deposition density in the non-realistic asymmetric and symmetric model shows a nearly alike deposition scenario in the left and right lung. The deposition concentration curve illustrates the DHS for the non-realistic model and which is the carinal angle. However, the deposition density is different in the right and left lung of the realistic model. The trapped particle density comparison at the left lung shows higher deposition for turbulent case compares to the laminar case. The aerosol particle deposition concentration for different turbulence models illustrate the DHS at the upper airways. The deposition density comparison curve reports higher deposition for LES turbulent model than *k*-ω turbulent model at the specific position of the airways. However, the overall DHS for different turbulence models against flow rates are found similar. The highly asymmetric bifurcating pattern of the realistic lung model stimulates the deposition pattern in both left and right lung. The precise understanding of the particle deposition concentration in the upper airways is important for better health risk assessment. The deposition density curve for the realistic CT-based model would sufficiently increase the knowledge of the particle deposition concentration at both lungs, which necessarily helps the pharmaceutical industry to design a better drug transport tool.

**Figure 12:**
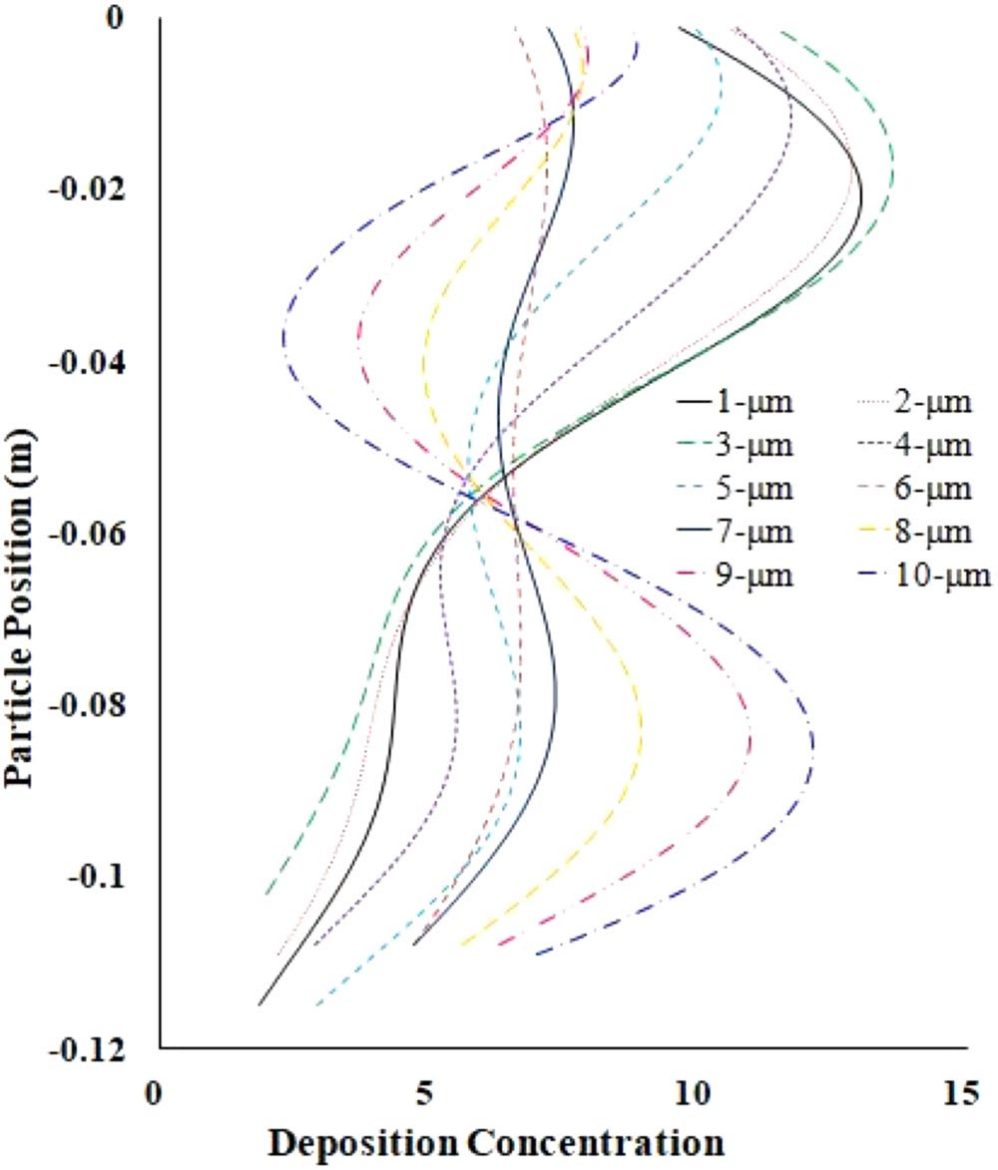
Deposition concentration comparison of polydisperse particles at 50 lpm (LES model).

The trapped particle density comparison of polydisperse particles in a high flow rate (50 lpm) is shown in Fig. 12. The trapped particle density comparison curve illustratess the DHS of polydisperse particles at the upper airways. The overall deposition comparison reports that larger particles are trapped mostly at the central bifurcating branches. The detail DHS for different diameter particles are investigated for the CT-based model. The overall DHS’s are calculated for LES turbulent modelling case. Table 4 illustrates the DHS for polydisperse particle at the trachea, and both lungs. The DHS table illustrates that there is no hot spot (HS) at the right and the left lung for lower flow rates (7.5 lpm); however a various HS is reported at the right and the left lung for higher flow rates (50 lpm). These particular findings will increase the understanding of the DHS in a realistic lung, which will eventually increase the DE of the zone-specific drug transport system. The pharmaceutical industry can design different drug delivery devices as the various DHS is observed for various diameter particles. After diagnosis, the patient can use particular drug delivery devices and it will reduce the pharmaceutical aerosol particle to the unwanted position of the respiratory airways.

**Table 4:** Polydisperse particle DHS at different flow rates. (No Hot Spot = NHS).

Diameter	Trachea		Right Lung		Left Lung	
	50 lpm	7.5 lpm	50 lpm	7.5 lpm	50 lpm	7.5 lpm
1	Upper part of trachea	NHS	G1	NHS	NHS	NHS
2	Upper part of trachea	NHS	G1	NHS	G2	NHS
3	Upper part of trachea	NHS	G1	NHS	G1	NHS
4	Trachea	NHS		NHS	G2	NHS
5	Trachea	NHS	G2	NHS		NHS
6	Trachea	Upper part of trachea	G2	NHS	G3	NHS
7	Trachea	Upper part of trachea	G2	NHS	G2	NHS
8	Trachea	Upper part of trachea	G2	NHS	Carinal Angle of G2	NHS
9	Trachea	Upper part of trachea	Upper bifurcation of G2	NHS	Carinal Angle of G2	NHS
10	Trachea and bifurcation wall	Upper part of trachea	G2 surface and Carinal Angle	NHS	G2-G3 Carinal Angle	NHS

**Figure 13:**
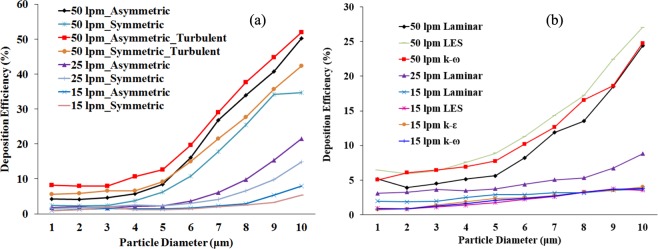
Polydisperse particle DE comparison for different flow rates and various anatomical models (**a**) symmetric and asymmetric model, and (**b**) realistic CT-based model.

The inclusive DE in the different anatomical models for a different viscous model, and the flow rates appear in Fig. 13. The DE in a non-realistic symmetric and asymmetric triple bifurcation model is shown in Fig. 13(a), and in a realistic CT-based model, shown in Fig. 13(b). The overall deposition pattern shows higher DE, regardless of particle diameter and flow rate. Figure 13(a) illustrates that the DE in an asymmetric triple bifurcation model was higher than in the symmetric bifurcation model. Moreover, during turbulent dispersion, the DE in an asymmetric triple bifurcation model was higher than in the non-turbulent case. Specifically, the negligible turbulent effect was observed on deposition with smaller diameter micro size particles (1 μm ≤ *d*_*p*_ ≤ 3 μm), for a higher flow rate (50 lpm). Furthermore, the turbulent effect on deposition in a non-realistic asymmetric and symmetric bifurcation model was insignificant for the micro-particles size from 1 μm ≤ *d*_*p*_ ≤ 6 μm in a flow rate less than 25 lpm. For lower flow rate, different turbulent model shows negligible deposition variation at the upper airways. However, for higher flow rate, the DE for LES model is found higher than the *k*-ω model.

The overall DE in a non-realistic airway model illustrates that the turbulent effect was significant for particles greater than 5-µm diameter. Turbulence effects on polydisperse micro-particles in a realistic lung airway were higher than for the laminar case. At higher flow rates (50 lpm), no turbulence effect was found for 1- µm diameter particles. The present CFD study also shows nearly similar DE for 9 µm–10 µm diameter particles at 50 lpm flow rate. With a lower flow rate, the turbulent effect on polydisperse micro-particle deposition in a realistic lung model was insignificant. However, the turbulent fluctuation influences the DE at higher flow rate for both non-realistic and realistic lung model.

**Figure 14:**
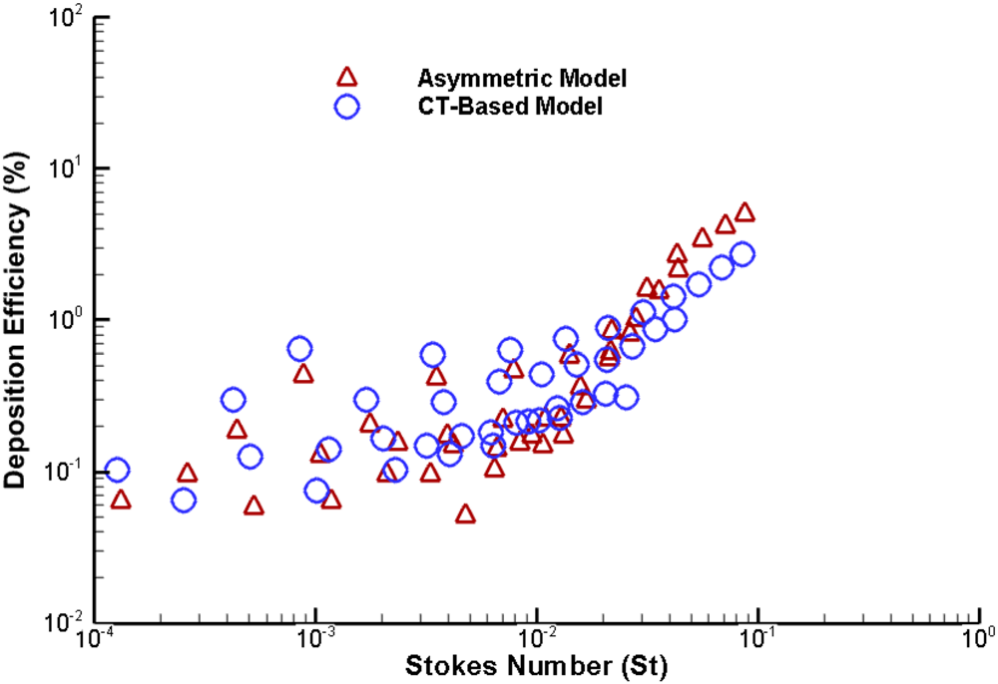
DE comparison for asymmetric non-realistic model and realistic model against Stokes number.

Figure 14 shows the DE comparison for a non-realistic asymmetric model and a CT-based realistic lung model against the Stokes number. Overall, DE vs. Stokes number shows an increasing trend, and DE increases with the increased Stokes number, which adequately supports the general phenomenon of the Stokes number. In case of low Stokes number, the aerosol particle can follow the air streamline, which indicates the lower deposition. On the contrary, for higher Stokes values, the particle deviates from the air streamline, especially when the fluid flow decelerates abruptly. In general, an increase of Stokes number means the inertial impaction will be dominant which is one of the leading mechanisms for micro-particle deposition. Figure 14 reports the deposition pattern for both anatomical models is proportional to the Stokes number.

## Conclusions

An advanced CFD model has been developed for polydisperse micro-particle TD in the upper bifurcating airways. To investigate turbulence effects on polydisperse aerosol particle TD, three different anatomical models are considered. The major conclusions from the present study are given below:

- The airflow distribution percentage in the right bifurcating airways of realistic geometry was higher than in the right lung of the non-realistic geometry. Specifically, flow distribution in the right bifurcating airways of the realistic geometry was 1.65 times greater than in the left lung. For non-realistic geometry, the flow distribution in the right bifurcating airways was 1.4 times than greater than the left lung.
- The respiratory anatomical model affects the DHS for realistic and non-realistic geometry. In the non-realistic anatomical model, the bifurcation area was the DHS, but in the realistic anatomical model, different DHS’s are reported for different flow rates.
- Turbulence influences the micro-particle deposition pattern in the upper bifurcating airways of the CT-based model. In the realistic anatomical model, a substantial number of polydisperse aerosols were deposited at the trachea, but no particles were deposited at the tracheal wall of the non-realistic model. Deposition at the tracheal duct due to turbulent fluctuation is observed higher than that of the non-turbulent case. Turbulence fluctuation influences the micro-particles deposition pattern for higher flow rate. However, the turbulence dispersion effects on micro-particle deposition are reported negligible.

The present CFD study performed an inclusive, polydisperse aerosol TD analysis in a realistic airway. The CFD model demonstrates the DE and DHS of various diameter aerosol at various parts of the pulmonary airways. Total flow rate distribution percentage in the right bifurcating airway and the left bifurcating airway was calculated to expand understanding of the resulting health hazard assessment and ventilation distribution of the diseased lung. The present model illustrates different DHS for a realistic model which would aid the zone-specific pharmaceutical aerosol transport and increase efficiency in zone-specific drug delivery. Inclusive airflow and polydisperse particle TD analysis will potentially assist the design of more proficient targeted drug transport tools. In a future study, the authors will consider a higher generation branching model for better prediction of monodisperse and polydisperse particle TD in a whole lung model.

### Ethics approval and consent to participate

This study was approved by the Prince Charles Hospital Human Research Ethics Committee (Approval number: HREC/16/QPCH/276) and the Queensland University of Technology Human Research Committee (Approval Number 1600000923).
